# Ascites re-compensation in HBV-related first decompensated cirrhosis after anti-viral therapy

**DOI:** 10.3389/fcimb.2022.1053608

**Published:** 2023-01-12

**Authors:** Mingyu Li, Zheng Zong, Xinmiao Xiong, Jing Fan, Huan Zhong, Na Liu, Wei Ye, Jisheng Jing

**Affiliations:** ^1^ Department of Liver Disease, The Second Hospital of Nanjing, Nanjing University of Chinese Medicine, Nanjing, China; ^2^ Department of Clinical Research Centre, The Second Hospital of Nanjing, Nanjing University of Chinese Medicine, Nanjing, China; ^3^ Department of Infectious Diseases, Jurong People’s Hospital, Jiangsu University, Zhenjiang, China

**Keywords:** decompensated cirrhosis, antiviral therapy, re-compensation of ascites, hepatitis B virus, liver cirrhosis

## Abstract

Effective antiviral therapy can significantly improve the long-term prognosis of HBV-related decompensated patients, and re-compensation may be achieved in part of the patients. To explore the re-compensation of ascites after HBV suppression and the risk factors, the clinical outcomes of 196 consecutive patients with HBV-related first decompensated cirrhosis of ascites treated with nucleos(t)ide analogue (NUC) were analyzed retrospectively. Among these patients, the median serum HBV DNA level was 5.0 (IQR, 3.0-6.0) log_10_ IU/mL before treatment. Most patients were given NUC with high barrier to resistance including ETV (152), TDF (1) and TAF (1). Initial combination of LAM plus ADV and LdT plus ADV was used in 41 patients and 1 patients, respectively. After NUC treatment, the percentage of patients with ascites regression was 77.6%, 81.4%, 70.5%, 93.8%, 80.8% at 12, 24, 36, 48, 60 months, respectively (P<0.001). The distribution of ascites severity showed that the patients’ ascites improved, with the proportion of no ascites and mild ascites gradually increased. The proportion of re-compensation of ascites defined as negative HBV DNA, improved liver function and ascites regression (off diuretics) was 59.7%, 70.0%, 52.3%, 59.4%, 46.2% at 12, 24, 36, 48, 60 months (P<0.001). The rate of ascites regression was higher in viral response (VR) cohort when compared with that in non-VR cohort. Univariate and multivariable analysis showed that level of serum ALT (OR:0.988, 95%CI, p=0.029) and load of serum HBV DNA (OR:0.78895%CI, p=0.044) at baseline were risk factors of re-compensation of ascites. This study demonstrated that antiviral therapy could reverse decompensation of ascites in HBV-related first decompensated cirrhosis and the level of ALT and HBV DNA were risk factors of ascites re-compensation.

## 1 Introduction

Hepatitis B virus (HBV) infection is a major cause of acute and chronic liver disease globally, which usually progresses to liver fibrosis, liver cirrhosis and hepatocellular carcinoma (HCC). Currently, despite the fact that preventive vaccines have been used for decades as well as the use of effective and well-tolerated viral suppressive medications since 1998, an updated estimate indicated that the total global HBV infection prevalence increased to 3.9%, corresponding to 292 million people globally, in 2016 (Nguyen et al., 2020). It is estimated that the prevalence rate of HBsAg positive in the general population in China is 5% ~ 6%, thus the chronic HBV infection is about 70 million cases, including 20 to 30 million cases of chronic Hepatitis B (CHB) (Liu et al., 2019).

The natural history of liver cirrhosis is characterized by an asymptomatic compensated phase followed by a decompensated phase (European Association for the Study of the Liver, 2018). Decompensated cirrhosis is a common cause of admissions, and these patients often have complex medical needs and are at high risk of in-hospital death. The typical clinical manifestations of decompensated cirrhosis include ascites, jaundice, hepatorenal syndrome, hepatic encephalopathy and variceal haemorrhage (Fontana, 2003). Among them, ascites is the most common manifestation of decompensation in cirrhosis, as 5% to 10% of patients with compensated cirrhosis per year develop to this complication (Ginés et al., 1987). Renal sodium retention due to the activation of sodium retention systems such as the renin-angiotensin-aldosterone system (RAAS) and sympathetic nervous system is the main cause of ascites. The resulting positive fluid equilibrium eventually causes the volume of the extracellular fluid to expand. The reduction of effective blood volume due to dilatation of visceral arteries is the main determinant of these changes (European Association for the Study of the Liver, 2018; Bernardi et al., 2015). The occurrence of ascites can affect social and economic life, which often leads to hospitalization, requires long-term treatment, and is the direct cause of further complications such as spontaneous bacterial peritonitis (SBP), restrictive ventilatory dysfunction, and abdominal hernia. The presence of ascites can predict a poor prognosis, with five-year survival rates decreasing from about 80% in compensated patients to about 30% in patients with decompensated cirrhosis of ascites (D'Amico et al., 2006).

Reversal of the decompensated state (defined as ascites re-compensation) in HBV-related decompensated cirrhosis of ascites treated with antiviral therapy is of significant prognostic significance. The concept of re-compensation implies that there is at least partial regression of the structural and functional changes of cirrhosis after removal of the aetiology of cirrhosis. At present, several studies showed that antiviral therapy had a significant effect on patients with decompensated HBV cirrhosis. Among them, a South Korean 10-year follow-up cohort study showed that MELD and CTP scores in patients with decompensated hepatitis B cirrhosis were decreased after effective antiviral therapy, suggesting that some patients might be re-compensated (Jang et al., 2018). The criteria of re-compensation and scoring system to predict re-compensation in patients with HBV-related decompensated cirrhosis were also explored in some studies (He et al., 2022; Kim et al., 2022; Wang et al., 2022). However, the definition of re-compensation had not been unified.

Ascites could present alone in 36% of patients and in combination with other complications in 37%. Therefore, it marked the transition to decompensation in 73% of cirrhotic patients. Moreover, it was also associated with worse clinical outcomes with higher mortality (D'Amico et al., 2022). As the clinical manifestations of patients with decompensated hepatitis B cirrhosis were complicated, it was still unclear whether different types of complications should be separately investigated or together. A multicenter retrospective case-control study included 553 patients with re-compensation and 3400 patients with acute decompensation with different causes. The regression analysis was conducted separately according to the different complications including gastrointestinal bleeding, bacterial infection, hepatic encephalopathy and ascites (Xu et al., 2021). In our study, we focused on ascites only as ascites was the most common reason of decompensation in cirrhosis patients. The re-compensation of ascites after HBV suppression and the factors associated with re-compensation of ascites in patients with HBV-related first decompensated cirrhosis of ascites were investigated.

## 2 Patients and methods

### 2.1 Study population

From September 2015 to November 2020, 265 patients with first decompensated HBV cirrhosis of ascites who were treated with antivirals were retrospectively screened in the Second Hospital of Nanjing. Clinical data of patients were collected using electronic medical record systems.

The inclusion criteria were as follows: (1) the reason for hospitalization was hepatitis B decompensated cirrhosis; (2) clinical symptoms of hospitalized patients included ascites; (3) HBV DNA>500 IU/mL; (4) patients were given antiviral drugs for the first time; (5) patients were followed up for at least 1 year. The exclusion criteria were as follows: (1) co-infection with other viral hepatitis; (2) death within 6 months; (3) less than one year of the follow-up time for the patients.

The study was approved by the ethical committee of the Second Hospital of Nanjing (2020-LY-kt043) and the requirement for informed consent was waived.

### 2.2 Diagnostic criteria, grading and definition

The presence of cirrhosis was determined based on liver histology findings, gross findings during surgery, or radiological findings of an irregular liver margin with ascites, varices, or thrombocytopenia (<10^5^ cells/mm^3^) (Kim et al., 2018). Decompensated cirrhosis was characterized by the development of overt clinical signs, including ascites, jaundice, hepatorenal syndrome, hepatic encephalopathy and variceal haemorrhage (Fontana, 2003).

Clinically, according to the amount of ascites, it could be divided into grade 1 (small amount), grade 2 (medium amount) and grade 3 (large amount). Grade 1 or a small amount of ascites: ascites could only be found through ultrasound examination; patients generally did not show abdominal distension; negative mobility dullness; under ultrasound, the ascites was located in each space with a depth < 3 cm. Grade 2 or moderate ascites: patients often had moderate abdominal distention and symmetrical abdominal heave; physical examination showed negative/positive mobility dullness; under ultrasound, the ascites flooded the intestine, but did not cross the middle abdomen, with a depth of 3 ~ 10 cm. Grade 3 or large ascites: abdominal distention was obvious; positive mobility dullness on physical examination; abdominal distention and even umbilical hernia might occur; under ultrasound, ascites occupied the entire abdominal cavity, and the middle abdomen was filled with ascites, with a depth of > 10 cm.

The primary endpoint was re-compensation of ascites. Clinically, some patients with decompensated HBV cirrhosis of ascites received effective antiviral therapy. Then, the patients’ condition was stable (>3 months) with improved liver function, serum HBV DNA negative (<500 IU/mL) and ascites regression after discontinuing diuretics, which were considered as re-compensation of ascites. The improved liver function was defined as improved serum total bilirubin (TBIL), alanine aminotransferase (ALT), aspartate aminotransferase (AST), albumin (ALB) combined with the values of TBIL, ALT, AST less than 2 ULN and ALB more than 28g/L. In this way, we attempted to achieve complete recovery in patients with liver dysfunction and cirrhotic ascites.

### 2.3 Treatment

All patients were treated with standard medications after diagnosis, such as antiviral therapy, symptomatic relief and supportive treatment. Patients were given the following antivirals for life: entecavir (ETV), lamivudine (LAM) plus adefovir dipivoxil (ADV), tebivudine (LdT) plus ADV, tenofovir fumarate (TDF) or tenofovir alafenamide fumarate (TAF).

### 2.4 Data collection

We collected demographic, interview, clinical and routine laboratory data on the first contact visit to the hospital for patients with first decompensation of HBV cirrhosis of ascites in an electronic medical record system. Demographic characteristics and interview data included age, gender, history of drug use, and other diseases (such as hypertension and diabetes). The laboratory data included white blood cells (WBC), red blood cells (RBC), hemoglobin, platelet count, TBIL, indirect bilirubin, levels of serum ALB, ALT, AST, creatinine, alkaline phosphatase (ALP), sodium, γ -glutamyltransferase (γ-GT), prothrombin activity, prothrombin time(PT), international normalized ratio (INR), fibrinogen (FIB), serum HBV DNA (detected by real-time fluorescent quantitative PCR with the lower limit of detection of 500 IU/mL). Imaging studies included ultrasound, computed tomography (CT), and magnetic resonance imaging (MRI). The laboratory and imaging data for the study were derived from evaluations performed within a month on the date closest to the baseline and follow-up.

### 2.5 Propensity score matching

We used logistic regression model to fit three relevant variables ascites grade, HBV DNA level, Child-Pugh grade for propensity score. Nearest neighbor matching (1:1 propensity matching) was used to establish a propensity matched cohort of treatment with either the ETV monotherapy group or the LAM+ADV combination therapy group.

### 2.6 Statistical analysis

Measurement data were expressed as mean ± standard deviation or median (quartile range [IQR]), and count data were expressed as number of cases or constituent ratio. When comparing the differences between the two groups, the *t*-test was used for continuous variables and the Fisher exact test or chi-square test was used for categorical variables. Based on clinical experience, a number of indicators were included in the univariate logistic regression to identify the factors associated with re-compensation of ascites, and then the significantly associated factors in univariate analysis (P<0.1) were included in multivariate analysis. The area under the ROC curve (AUC) with 95% confidence intervals (CI) was used to assess the predictive accuracy of the occurrence of re-compensation after at least 1 year of NUC treatment.

All statistical analyses were conducted using SPSS 22.0 and GraphPad Prism8.0.2. Among this study, two-tailed P<0.05 was considered statistically significant.

## 3 Results

### 3.1 Demographic characteristics

**Figure 1:**
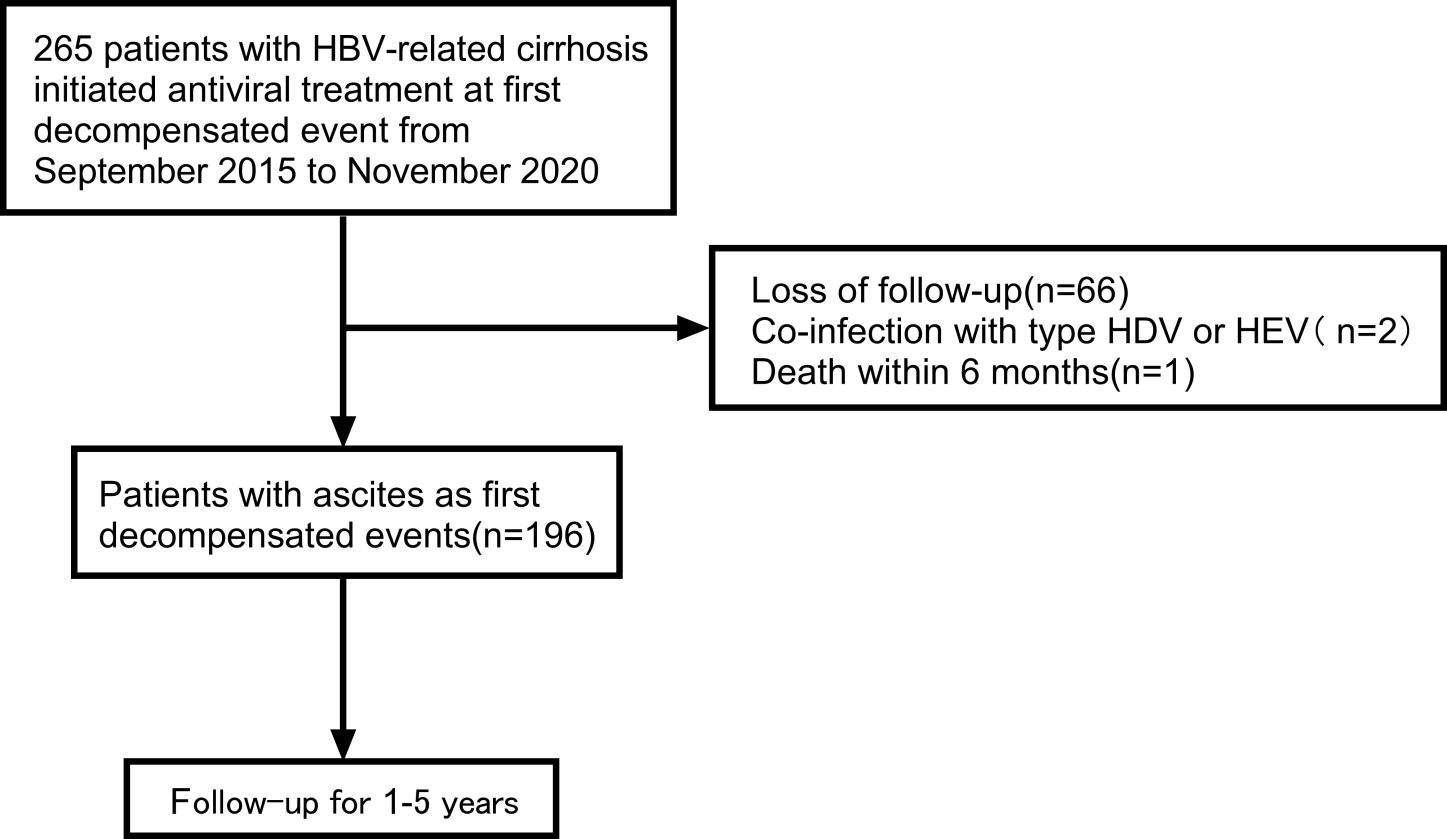
Flow chart of the patient selection process. HBV, viral hepatitis B; HDV, viral hepatitis D; HEV, viral hepatitis E.

A total of 265 patients were enrolled in the study. Overall, 69 patients were excluded according to the exclusion criteria. **Figure 1** presents the flow diagram of patient selection. 196 patients were included for the final analysis. The 196 patients were followed for 1-5 years with median follow-up time 13.58 months and the flow chart of the total cohort was shown in **Figure 1**. The baseline characteristics of the total cohort of 196 patients were shown in **Table 1**. Among these patients, males were predominated (69.9%) and the mean age was 52.0 years. The first decompensated event was ascites (100%). At the beginning of NUC treatment, the median serum HBV DNA level was 5.0 (IQR, 3.0-6.0) log_10_ IU/mL. The percentage of Child-pugh class A, B, C was 2.0%, 47.4%, 50.5%, respectively (**Table 1**). Most patients were given NUC with high barrier to resistance including ETV (152/196), TDF (1/196) and TAF (1/196). Initial combination of LAM plus ADV and LdT plus ADV was used in 41 patients and 1 patient, respectively (**Table S1**).

**Table 1:** Baseline characteristics of the patients in the total cohort.

Varibles	Total (n=196)
Age, y	52.0 ± 10.3
Male, n	137 (69.9)
Diabetes mellitus, n	20 (10.2)
Hypertension, n	16 (8.2)
WBC, ×10^9^/L	3.6 (2.7-5.1)
RBC, ×10^12^/L	3.6 ± 0.6
Hemoglobin, g/L	115 (102.3-127.8)
Platelet, ×10^9^/L	58.5 (41.5-90.8)
Total bilirubin, μmol/L	37.8 (23.5-67.5)
Indirect bilirubin, µmol/L	19.1 (13.0-30.0)
Albumin, g/L	30.9 (27.0-34.7)
ALT, IU/L	43.7 (31.4-123.7)
AST, IU/L	64.2 (42.8-131.2)
Creatinine, μmol/L	67 (57-78)
ALP, IU/L	108.5 (85.9-140.4)
Sodium, mmol/L	140 (138.1-141.8)
γ-GT, U/L	63.8 (33.1-114.5)
INR	1.4 (1.3-1.7)
Prothrombin activity, %	50.4 (42.1-58.5)
Prothrombin time (s)	15.2 (16.5-19.0)
FIB, g/L	1.4 (1.1-1.7)
HBV DNA, log_10_ IU/mL	5.0 (3.0-6.0)
Child-Pugh class	
A	4 (2.0)
B	93 (47.4)
C	99 (50.5)
MELD score	11.0 (8.0-15.0)

RBC, red blood cell count; WBC, white blood cell count; ALT, alanine aminotransferase; AST, aspartate aminotransferase; ALP, alkaline phosphatase; γ-GT, γ-glutamyl transferase; INR, international normalized ratio; FIB, fibrinogen; MELD, model for end-stage liver disease.

### 3.2 Clinical outcomes

**Figure 2:**
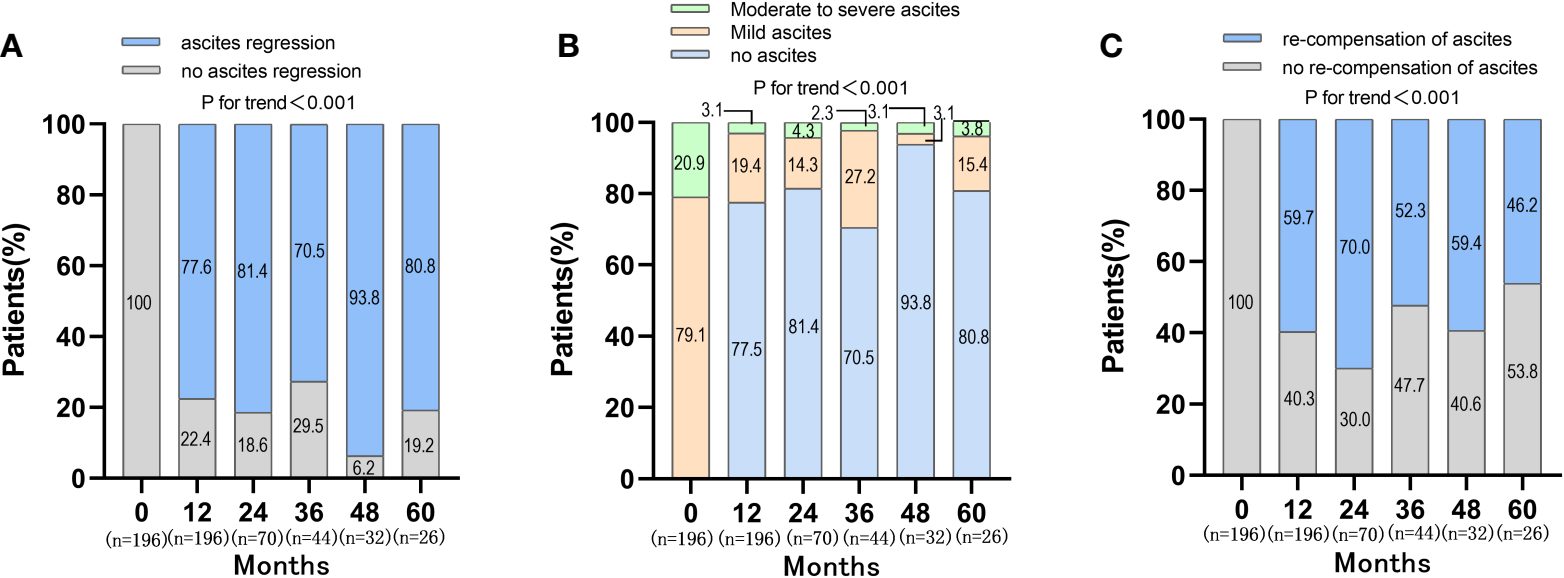
Changes of ascites and re-compensation of ascites in patients with first decompensated HBV cirrhosis of ascites after NUC treatment. **(A)** Changes of ascites in patients after NUC treatment at baseline, 12, 24, 36, 48 and 60 months; **(B)** Distribution of ascites severity in patients after NUC treatment at baseline, 12, 24, 36, 48 and 60 months; **(C)** Re-compensation of ascites in patients after NUC treatment at baseline, 12, 24, 36, 48 and 60 months.

During the follow-up period, the proportion of patients with ascites regression was 77.6%, 81.4%, 70.5%, 93.8%, 80.8% at 12, 24, 36, 48, 60 months, respectively (P<0.001) (**Figure 2**). The distribution of ascites severity showed that the patients’ ascites improved, with the proportion of no ascites and mild ascites gradually increased and the proportion of moderate to severe ascites decreased (P<0.001) (**Figure 2**). The proportion of patients with moderate to severe ascites was 20.9%, 3.1%, 4.3%, 2.3%, 3.1%, 3.8% at baseline, 12, 24, 36, 48, 60 months, respectively. When the re-compensation of ascites was defined as HBV DNA negative (<500 IU/mL), no diuretics, liver function improvement and ascites regression, the proportion of patients with re-compensation of ascites was 59.7%, 70.0%, 52.3%, 59.4%, 46.2% at 12, 24, 36, 48, 60 months (P<0.001) (**Figure 2**). Then, the baseline characteristics were compared between the re-compensation of ascites group and no re-compensation of ascites group. There were no obvious differences for the percentage of ETV therapy, HBV DNA level, ALT and AST at baseline (**Table S2**).

To assess the effect of antiviral treatment on biochemical response in patients with decompensated HBV cirrhosis of ascites, total bilirubin, ALT, and AST were measured and compared at baseline and follow-up period (**Supplementary Figure S1**). The routinely tested parameters of total bilirubin (TB), ALT, AST improved after initiation of antiviral therapy (**Figure S1**). The rate of ALT normalization in ETV monotherapy group was higher compared with that in the LAM+ADV combination therapy group after 12-month treatment (**Table S1**). However, after Propensity Score Matching, the ALT normalization rates were similar betweeen these two groups (**Figure S2A**).

### 3.3 Virological response

**Figure 3:**
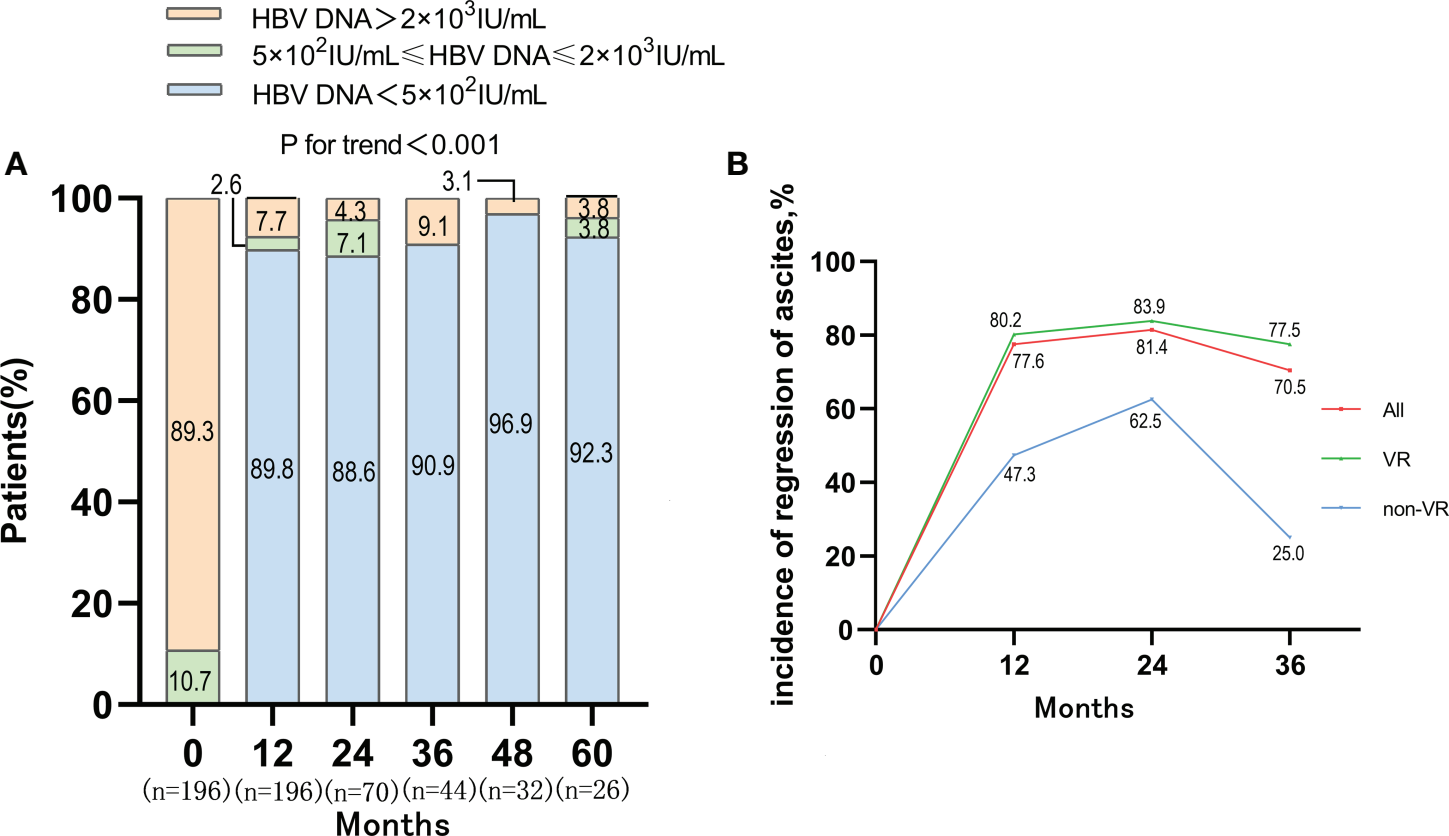
VR and the incidence of regression of ascites in VR and non-VR group in patients with first decompensated HBV cirrhosis of ascites after NUC treatment. **(A)** Dynamic change of HBV DNA level after antiviral treatment; **(B)** Regression of ascites occurred with incidences of 77.6%, 81.4% and 70.5% in the whole cohort at 1, 2 and 3 years, and the VR cohort achieved a higher re-compensated rate than the non VR cohort.

After antiviral treatment, the proportion of patients with high level of HBV DNA (>2×10^3^ IU/mL) was decreased dramatically. The proportion of patients with HBV DNA <500 IU/mL increased to 89.8%, 88.6%, 90.9%, 96.9%, 92.3% at 12, 24, 36, 48, 60 months respectively (P<0.001) (**Figure 3**). The virological response (VR) rates of ETV monotherapy therapy and LAM+ADV combination therapy were 93.42% and 78.05% after 12-month antiviral treatments, respectively (P<0.05) (**Table S1**). After Propensity Score Matching, the virological response rates of these two types of antiviral therapy were 95.12% and 80.05%, respectively (P<0.05) (**Figure S2B**).

The incidence of ascites regression was 77.6%, 81.4% and 70.5% at 1-year, 2-year and 3-year, respectively. Among patients who achieved VR, the incidence of ascites regression was 80.2%, 83.9%, and 77.5% at 1-year, 2-year and 3-year respectively. Among the 20 patients who failed to achieve VR, the incidence of ascites regression was only 47.3%, 62.5%, and 25% at 1-year, 2-year, and 3-year, respectively. The rate of ascites regression in VR cohort was higher than that in non-VR cohort, which suggested that VR might be one of the determinants of disappearance of ascites in patients with first decompensated HBV cirrhosis of ascites after antiviral treatment (**Figure 3**). There was no obvious difference between the rate of ascites regression in ETV monotherapy group and that in LAM+ADV combination therapy at 1-year (**Figure S2C**).

### 3.4 Factors associated with re-compensation of ascites

In the entire cohort, univariate analysis based on the competitive risk model revealed a number of pre-treatment variables associated with re-compensation of ascites including hemoglobin, bilirubin, ALT, AST, ALP, PT (INR), HBV DNA, MELD score. These significant variables were incorporated into the multivariable analysis. The results showed that ALT (OR:0.988, 95%CI, p=0.029) and HBV DNA (OR:0.788, 95%CI, p=0.044) were risk factors of re-compensation of ascites (**Table 2**).

**Table 2:** Factors associated with re-compensation of ascites following NUC treatment in the total cohort.

Factors	Univariate analyses	P value	Multivariate analyses	P value
	OR (95% CI)		OR(95% CI)	
Male, n	1.032 (0.520-1.032)	0.929		
Diabetes mellitus, n	0.677 (0.255-1.799)	0.434		
Hypertension, n	0.454 (0.160-1.288)	0.138		
WBC, ×10^9^/L	0.904 (0.786-1.040)	0.157		
RBC, ×10^12^/L	0.825 (0.506-1.345)	0.44		
Hemoglobin, g/L	0.982 (0.968-0.997)	0.018	0.989 (0.972-1.005)	0.185
Platelet, ×10^9^/L	0.996 (0.989-1.004)	0.345		
Total bilirubin, µmol/L	0.991 (0.983-0.998)	0.015	0.996 (0.986-1.005)	0.358
Albumin, g/L	1.004 (0.946-1.066)	0.893		
ALT, IU/L	0.993 (0.988-0.998)	0.006	0.988 (0.978-0.999)	0.029
AST, IU/L	0.994 (0.990-0.999)	0.012	1.008 (0.999-1.017)	0.089
Creatinine, μmol/L	0.995 (0.982-1.009)	0.521		
ALP, IU/L	0.993 (0.985-1.000)	0.066	0.996 (0.988-1.004)	0.322
Sodium, mmol/L	1.007 (0.969-1.047)	0.727		
Prothrombin activity, %	1.014 (0.991-1.037)	0.242		
Prothrombin time, s	0.900 (0.809-1.001)	0.052	0.869 (0.733-1.030)	0.104
FIB, g/L	1.108 (0.664-1.851)	0.694		
HBV DNA, log_10_ IU/mL	0.767 (0.624-0.943)	0.012	0.788 (0.625-0.994)	0.044
Child-Pugh class	0.941 (0.783-1.132)	0.521		
MELD score	0.925 (0.867-0.986)	0.017	1.024 (0.905-1.159)	0.71

RBC, red blood cell count; WBC, white blood cell count; ALT, alanine aminotransferase; AST, aspartate aminotransferase; ALP, alkaline phosphatase; FIB, fibrinogen; MELD, model for end-stage liver disease.

**Figure 4:**
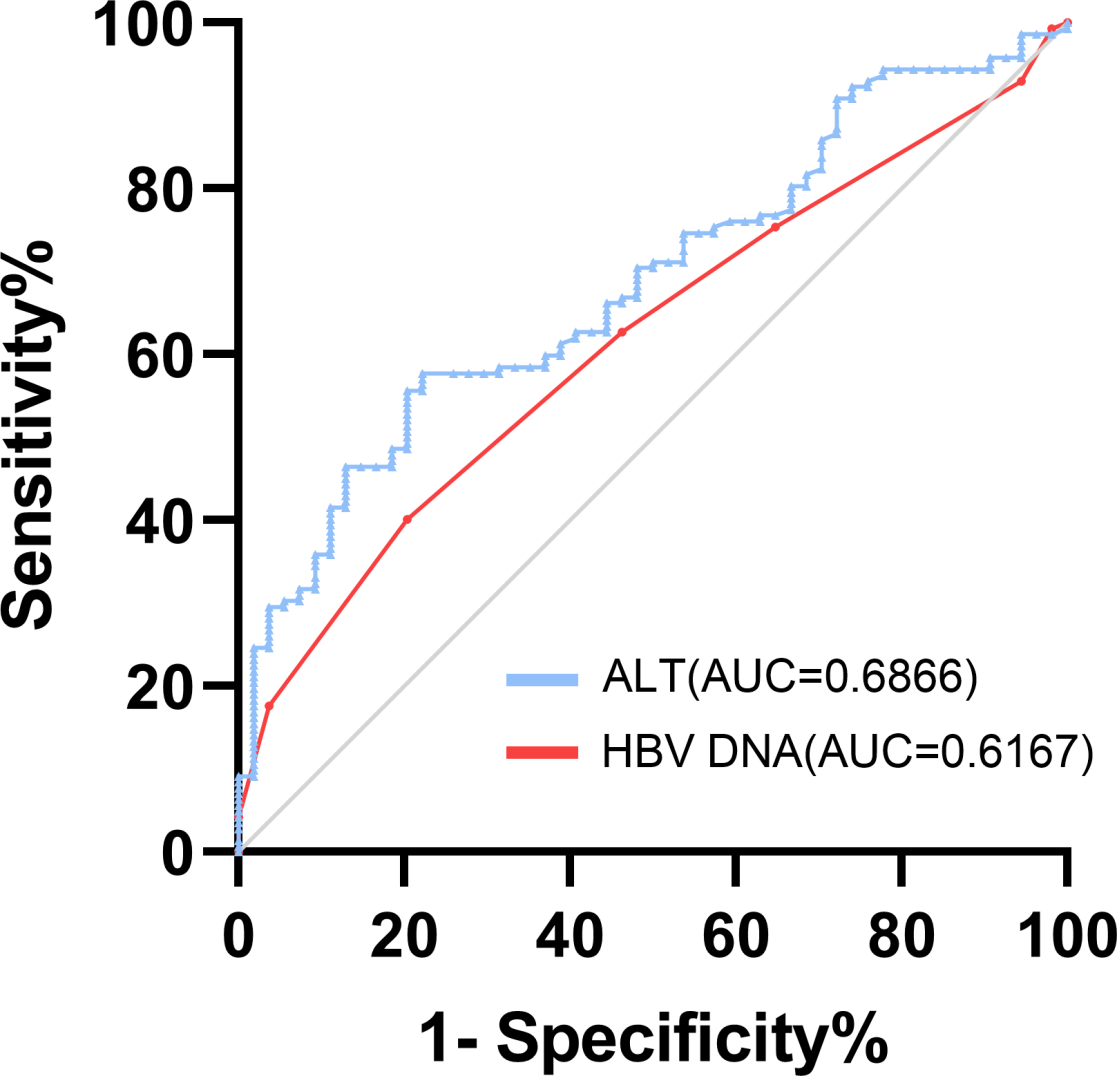
ROC curve analysis used to calculate the best cut-off point of ALT and HBV DNA level for predicting the occurrence of re-compensation of ascites in patients with HBV-related first decompensated cirrhosis of ascites. Best cut-off points of ALT and HBV DNA levels as 46.1 IU/L (sensitivity%,57.70% and specificity%, 77.80%) and 5 log_10_ IU/mL (sensitivity%,33.35% and specificity%, 81.95%). The area under curve (AUC) of ALT and HBV DNA were 0.6866 [95% confidence interval (95%CI):0.609-0.764, P<0.001] and 0.6167 [95% confidence interval (95%CI):0.534-0.699, P=0.012], respectively. AUC, area under the curve; ROC, receiver operating characteristic.

Then, The ROC curve analysis revealed that when setting the cut-off value of ALT<46.1 IU/L and the cut-off value of HBV DNA<5 log_10_ IU/mL, the area under curve (AUC) of ALT and HBV DNA were 0.6866 [95% confidence interval (95%CI):0.609-0.764, P<0.001] and 0.6167 [95% confidence interval (95%CI):0.534-0.699, P=0.012], respectively. (**Figure 4**) The sensitivity and specificity of ALT were 57.70% and 77.80%, with positive predictive value of 36.52%, negative predictive value of 25.93%, + LR of 0.74 and - LR of 0.57 and the sensitivity and specificity of HBV DNA were 33.35% and 81.95%, with positive predictive value of 57.02%, negative predictive value of 25.61%, + LR of 1.85 and - LR of 0.81.

## 4 Discussion

Antiviral therapy for chronic hepatitis B virus prevents progression to clinical complications associated with decompensation of cirrhosis and hepatocellular carcinoma (Marcellin et al., 2013). Several studies have generally focused on the risk of mortality and hepatocellular carcinoma, development of decompensation, and identification of related factors (Kim et al., 2022). However, considering the corrective effect of NUCs on liver function and fibrosis, it is also important to elucidate its potential effect on the reversal of decompensated cirrhosis complications. Re-compensation is a special phase of decompensated liver cirrhosis. After effective treatment over time, liver function enables the patient to perform daily activities without the complications associated with decompensated cirrhosis (Zhao et al., 2020). To date, there has been a lack of a comprehensive assessment to identify patients with a “re-compensated advantage”. In this study, we observed the occurrence of ascites re-compensation after anti-viral therapy in patients with HBV-related first decompensated cirrhosis and analyzed factors associated with re-compensation of HBV-related decompensated cirrhosis of ascites. Most patients could achieve ascites re-compensation after effective NUC therapy. It was also demonstrated that ALT and HBV DNA at baseline were predictors of re-compensation of the decompensated HBV-related cirrhosis of ascites.

The objective of treatment of decompensated cirrhosis was to improve liver function, reduce secondary decompensated events, prolong the survival time and even achieve the re-compensation. Clinically, the definition of re-compensation had not been unified. Simply, it should include persistent absence of complications (He et al., 2019). In Baveno VII concensus, the criteria of re-compensation should include removal/suppression/cure of the primary aetiology of cirrhosis; resolution of ascites (off diuretics), encephalopathy (off lactulose/rifaximin) and absence of recurrent variceal haemorrhage (for at least 12 months); stable improvement of liver function (albumin, INR, bilirubin) (De Franchis et al., 2022). However, these criteria did not define the cut-off values for stable improvement of liver function tests. Recent research suggested that MELD score <10 and/or liver function tests within Child-Pugh A would be used as a criterion for stable improvement of liver function tests (Wang et al., 2022). In this study, the values of TBIL, ALT, AST and ALB were used as the criteria for stable improvement of liver function, which could be easily obtained from the liver function test. There were 77.6%, 81.4%, 70.5%, 93.8%, 80.8% of patients achieving ascites regression at 12, 24, 36, 48, 60 months after anti-viral therapy in HBV-related first decompensated cirrhosis of ascites, respectively. However, when the ascites re-compensation was defined as disappeared ascites (off diuretics), HBV DNA negative (<500 IU/mL) and liver function improvement, there were only 59.7%, 70.0%, 52.3%, 59.4%, 46.2% patients at 12, 24, 36, 48, 60 months achieving the objective, respectively.

In the context of effective etiological control of cirrhosis, multiple studies have shown that patients in compensated cirrhosis stage may reverse or even return to the non-cirrhotic stage (Marcellin et al., 2013; Jang et al., 2018; Rong et al., 2022). Some patients with decompensated hepatitis B cirrhosis could achieve improvements in liver function and a reduction in portal-hypertension-related complications after effective antiviral therapies (Singal and Fontana, 2012). A retrospective study screened 311 patients with decompensated HBV cirrhosis and Child-Pugh scores of 7 or higher at baseline and re-compensation was defined as the restoration of cirrhosis status to a Child-Pugh score of 5, which was maintained for at least 2 months. Then, re-compensation occurred in 57.2% and 66.7% of the subjects in the derivation and validation cohorts after NUC therapies (Kim et al., 2022). In this study, the liver function and HBV DNA levels were significantly improved after NUC therapy for more than 1 year. Symptom of ascites was also relieved dramatically and the percentage of mild, moderate to severe ascites decreased from 79.1%, 20.9% to 19.4%, 3.1% separately after 1 year of NUC therapy. Further, our data showed that the regression of ascites was related to the viral response. The percentage of patients without ascites was higher in viral response group compared with non-viral response group after antiviral therapy (80.2% vs 47.3%, 83.9% vs 62.5%, 77.5% vs 25% at year 1, 2, 3, separately), which was similar to previous research (Hanafy et al., 2019). Our results strongly supported the possibility of ascites re-compensation after effective antiviral therapy for patients with HBV-related first-time decompensated cirrhosis of ascites.

In this study, most patients were given NUC with high barrier to resistance including ETV, TDF and TAF. Initial combination of LAM plus ADV was used in 20.9% patients as the combination treatment approach was also tried in some chinese hospitals 7 years ago. There were also some studies exploring the efficacy of LAM combined with ADV versus ETV monotherapy in patients with hepatitis B associated decompensated cirrhosis. The reduction of ALT levels, HBV DNA levels, the rate of ALT normalization, undetectable HBV DNA, HBV e antigen (HBeAg) loss, HBeAg seroconversion and mortality were similar between the two groups (Lian et al., 2013; Peng et al., 2014). Our data showed that both of the virological response rate and ALT normalization rate were higher after 12-month therapy in ETV monotherapy group compared with combination group. However, after propensity matching analysis there were no obvious differences for the ALT normalization rate between these two groups. ETV monotherapy might be a better choice for patients with HBV-related decompensation compared with LAM plus ADV. Most patients taking LAM plus ADV had switched to the first-line regimens as the price of ETV and TDF had been decreased dramatically in China.

The age and gender distribution is related to the severity of liver diseases. The average age was 52 years and the proportion of male patients was up to 69.9% as all of the included patients were in the stage of decompensation in this study, which suggested the disease progression was more likely in more advanced age and males might be more likely to have severe HBV-related diseases, consistent with prior findings. A study showed that the sex ratio (male/female) increased with severity of liver diseases: 1.2 in HBV carrier, 1.8 in chronic hepatitis, 3.3 in HBV-related cirrhosis and 3.9 in HBV-related HCC (Liu et al., 2022). HBV genotype plays a role in the progression of HBV-related liver cirrhosis and HCC as well as response to antiviral therapy (Lin and Kao, 2017). Genotype B or C are most prevalent in China. Some studies found that HBV genotype B is associated with a slower rate of progression to cirrhosis, and a lower rate of HCC development compared with genotype C. A significantly higher incidence of HCC had been reported in persons infected with genotypes C or F compared with the others (Ching et al., 2016; Terrault et al., 2018; McMahon et al., 2021). The genotype was not detected routinely in this study, thus its relationship with re-compensation of ascites could not be evaluated, which still need to be further explored in future research.

The significant differences in clinical outcomes based on the presence or absence of re-compensation highlighted the importance and necessity of predicting re-compensation in the treatment of patients with decompensated cirrhosis of ascites receiving antiviral therapy against infection (D'Amico, 2014; Vinaixa et al., 2017; Hanafy et al., 2019). In this study, we identified two pre-treatment variables, ALT and HBV DNA, that were associated with re-compensation of HBV-associated decompensated cirrhosis of ascites treated with NUC therapy. In our study, the rate of undetected HBV DNA patients increased after antiviral treatment. However, it decreased from month 48 (96.9%) to month 60 (92.3%), which might be related with the limited number of patients in month 60. VR was associated with the ascites regression, which had also been demonstrated by another research (Kim et al., 2022). The HBV DNA level at baseline was one of the variables in the multivariable analysis, which was in accord of the results about VR, as patients with higher level of HBV DNA might face more difficulty to achieve VR. Higher ALT levels before treatment in patients with hepatitis B, as markers of increased inflammatory activity for liver necrosis, was related to the worse clinical outcomes, which suggested that biochemical response might be also important for the ascites re-compensation after NUC therapy in HBV-associated decompensated cirrhosis of ascites. Besides, an upsurge of serum HBV DNA and hepatitis B surface antigen levels usually precedes the abrupt rise of ALT levels, which could explain the synergistic effect between level of HBV DNA and ALT (Chang and Liaw, 2014; Chien and Liaw, 2022). Further, it was found that the best cut-off value of ALT and HBV DNA levels was chosen at 46.1 IU/L and 5 log_10_ IU/mL by ROC curve analysis.

The advantage of this study was that the ascites re-compensation was strictly defined as disappeared ascites (off diuretics), HBV DNA negative (<500 IU/mL) and liver function improvement according to the Baveno VII consensus and the occurrence was explored in patients with HBV-related first decompensation cirrhosis of ascites. However, there were several limitations to our study. First, this study was a retrospectively designed, thus inherent limitations of selection bias were inevitable. Patients who had less than 1 year follow up and who had death within 6 months were excluded from the criteria might overestimate the prognosis. Second, as our study was based on a limited number of patients from a single medical center, the factors associated with ascites re-compensation in HBV-related decompensated cirrhosis of ascites was unique, there was no other cohorts available for external validation. Besides, the sample size was too small in the 60-months group, which might affect the reliability of conclusion. Third, the low limit of HBV DNA was 500 IU/mL in this study and the proportion of patients with VR might change if the HBV DNA assay with 10 IU/mL detection limit was used.

In conclusion, this study showed that antiviral therapy could reverse ascites and the level of ALT and HBV DNA were risk factors of ascites re-compensation after antiviral therapy in patients with HBV-related decompensated cirrhosis of ascites. Overall, this study provided important information on the changes of ascites after HBV suppression in patients with decompensated HBV cirrhosis and shed light on the future treatment and care in HBV cirrhosis patients. These results may warrant further validation in prospective, multicenter, large-scale trials in future.

## Data availability statement

The original contributions presented in the study are included in the article/**Supplementary Material**. Further inquiries can be directed to the corresponding authors.

## Ethics statement

The studies involving human participants were reviewed and approved by the ethical committee of the Second Hospital of Nanjing. Written informed consent for participation was not required for this study in accordance with the national legislation and the institutional requirements. Written informed consent was not obtained from the individual(s) for the publication of any potentially identifiable images or data included in this article.

## Author contributions

JJ and WY designed the study. ML, ZZ and XX implemented this research. HZ and NL collected medical records. ML, JF and WY drafted the manuscript. All the authors participated in the revision of the manuscript. All authors contributed to the article and approved the submitted version.

## References

[B1] Bernardi M. Moreau R. Angeli P. Schnabl B. Arroyo V. (2015). Mechanisms of decompensation and organ failure in cirrhosis: From peripheral arterial vasodilation to systemic inflammation hypothesis. J. Hepatol. 63, 1272–1284. doi: 10.1016/j.jhep.2015.07.004 26192220

[B2] Chang M. L. Liaw Y. F. (2014). Hepatitis b flares in chronic hepatitis b: pathogenesis, nsatural course, and management. J. Hepatol. 61, 1407–1417. doi: 10.1016/j.jhep.2014.08.033 25178562

[B3] Chien R. N. Liaw Y. F. (2022). Current trend in antiviral therapy for chronic hepatitis b. Viruses 14, 434 doi: 10.3390/v14020434 35216027 PMC8877417

[B4] Ching L. K. Gounder P. P. Bulkow L. Spradling P. R. Bruce M. G. Negus S.. (2016). Incidence of hepatocellular carcinoma according to hepatitis b virus genotype in Alaska native people. Liver Int. 36, 1507–1515. doi: 10.1111/liv.13129 27009849 PMC5021564

[B5] D'Amico G. (2014). The clinical course of cirrhosis. population based studies and the need of personalized medicine. J. Hepatol. 60, 241–242. doi: 10.1016/j.jhep.2013.10.023 24211741

[B6] D'Amico G. Bernardi M. Angeli P. (2022). Towards a new definition of decompensated cirrhosis. J. Hepatol. 76, 202–207. doi: 10.1016/j.jhep.2021.06.018 34157322

[B7] D'Amico G. Garcia-Tsao G. Pagliaro L. (2006). Natural history and prognostic indicators of survival in cirrhosis: a systematic review of 118 studies. J. Hepatol. 44, 217–231. doi: 10.1016/j.jhep.2005.10.013 16298014

[B8] De Franchis R. Bosch J. Garcia-Tsao G. Reiberger T. Ripoll C. Baveno VII Faculty (2022). Baveno VII - renewing consensus in portal hypertension. J. Hepatol. 76, 959–974. doi: 10.1016/j.jhep.2021.12.022 35120736 PMC11090185

[B21] European Association for the Study of the Liver (2018). EASL clinical practice guidelines for the management of patients with decompensated cirrhosis. J. Hepatol. 69, 406–460. doi: 10.1016/j.jhep.2018.08.009 29653741

[B9] Fontana R. J. (2003). Management of patients with decompensated HBV cirrhosis. Semin. Liver Dis. 23, 89–100. doi: 10.1055/s-2003-37591 12616454

[B10] Ginés P. Quintero E. Arroyo V. Terés J. Bruguera M. Rimola A.. (1987). Compensated cirrhosis: natural history and prognostic factors. Hepatology 7, 122–128. doi: 10.1002/hep.1840070124 3804191

[B11] Hanafy A. S. Bassiony M. A. Basha M. A. A. (2019). Management of HCV-related decompensated cirrhosis with direct-acting antiviral agents: who should be treated? Hepatol. Int. 13, 165–172. doi: 10.1007/s12072-019-09933-8 30758786

[B13] He Z. Y. Wang B. Q. You H. (2019). [Reversal of cirrhotic decompensation: re-compensation]. Zhonghua Gan Zang Bing Za Zhi 27, 915–918. 10.3760/cma.j.issn.1007-3418.2019.12.002 31941252 PMC12814703

[B12] He Z. Zhou J. Tian Y. Wu S. Sun Y. Ou X.. (2022). Two-year free of complications during antiviral therapy predicts stable re-compensation in immediate-treatment HBV-related decompensated cirrhosis. Scand. J. Gastroenterol., 1–9. doi: 10.1080/00365521.2022.2132532 36227688

[B14] Jang J. W. Choi J. Y. Kim Y. S. Yoo J. J. Woo H. Y. Choi S. K.. (2018). Effects of virologic response to treatment on short- and long-term outcomes of patients with chronic hepatitis b virus infection and decompensated cirrhosis. Clin. Gastroenterol. Hepatol. 16, 1954–1963 e3. doi: 10.1016/j.cgh.2018.04.063 29753085

[B15] Kim T. H. Ku D. H. Um S. H. Lee H. A. Park S. W. Chang J. M.. (2018). How can we improve the performance of model for end-stage liver disease sodium score in patients with hepatitis b virus-related decompensated liver cirrhosis commencing antiviral treatment? J. Gastroenterol. Hepatol. 13, 1641-1648 doi: 10.1111/jgh.14128 29462844

[B16] Kim T. H. Um S. H. Lee Y. S. Yim S. Y. Jung Y. K. Seo Y. S.. (2022). Determinants of re-compensation in patients with hepatitis b virus-related decompensated cirrhosis starting antiviral therapy. Aliment Pharmacol. Ther. 55, 83–96. doi: 10.1111/apt.16658 34662436

[B17] Lian J. S. Zeng L. Y. Chen J. Y. Jia H. Y. Zhang Y. M. Xiang D. R.. (2013). *De novo* combined lamivudine and adefovir dipivoxil therapy vs entecavir monotherapy for hepatitis b virus-related decompensated cirrhosis. World J. Gastroenterol. 19, 6278–6283. doi: 10.3748/wjg.v19.i37.6278 24115827 PMC3787360

[B18] Lin C. L. Kao J. H. (2017). Natural history of acute and chronic hepatitis b: The role of HBV genotypes and mutants. Best Pract. Res. Clin. Gastroenterol. 31, 249–255. doi: 10.1016/j.bpg.2017.04.010 28774406

[B19] Liu J. Liang W. Jing W. Liu M. (2019). Countdown to 2030: eliminating hepatitis b disease, China. Bull. World Health Organ 97, 230–238. doi: 10.2471/BLT.18.219469 30992636 PMC6453311

[B20] Liu M. Li L. Zhao J. Ungvari G. S. Ng C. H. Duan Z.. (2022). Gender differences in demographic and clinical characteristics in patients with HBV-related liver diseases in China. PeerJ 10, e13828. doi: 10.7717/peerj.13828 35959480 PMC9359133

[B22] Marcellin P. Gane E. Buti M. Afdhal N. Sievert W. Jacobson I. M.. (2013). Regression of cirrhosis during treatment with tenofovir disoproxil fumarate for chronic hepatitis b: a 5-year open-label follow-up study. Lancet 381, 468–475. doi: 10.1016/S0140-6736(12)61425-1 23234725

[B23] McMahon B. J. Nolen L. D. Snowball M. Homan C. Negus S. Roik E.. (2021). HBV genotype: A significant risk factor in determining which patients with chronic HBV infection should undergo surveillance for HCC: The hepatitis b Alaska study. Hepatology 74, 2965–2973. doi: 10.1002/hep.32065 34292609 PMC10929546

[B24] Nguyen M. H. Wong G. Gane E. Kao J. H. Dusheiko G. (2020). Hepatitis b virus: Advances in prevention, diagnosis, and therapy. Clin. Microbiol. Rev. 33, e00046-19 doi: 10.1128/CMR.00046-19 PMC7048015 32102898

[B25] Peng H. Liu J. Yang M. Tong S. Yin W. Tang H.. (2014). Efficacy of lamivudine combined with adefovir dipivoxil versus entecavir monotherapy in patients with hepatitis b-associated decompensated cirrhosis: A meta-analysis. J. Clin. Pharmacol. 54, 189–200. doi: 10.1002/jcph.181 24105676

[B26] Rong G. Chen Y. Yu Z. Li Q. Bi J. Tan L.. (2022). Synergistic effect of biejia-ruangan on fibrosis regression in patients with chronic hepatitis b treated with entecavir: A multicenter, randomized, double-blind, placebo-controlled trial. J. Infect. Dis. 225, 1091–1099. doi: 10.1093/infdis/jiaa266 32437567 PMC8921993

[B27] Singal A. K. Fontana R. J. (2012). Meta-analysis: oral anti-viral agents in adults with decompensated hepatitis b virus cirrhosis. Aliment Pharmacol. Ther. 35, 674–689. doi: 10.1111/j.1365-2036.2011.04990.x 22257108

[B28] Terrault N. A. Lok A. S. F. Mcmahon B. J. Chang K. M. Hwang J. P. Jonas M. M.. (2018). Update on prevention, diagnosis, and treatment of chronic hepatitis b: AASLD 2018 hepatitis b guidance. Hepatology 67, 1560–1599. doi: 10.1002/hep.29800 29405329 PMC5975958

[B29] Vinaixa C. Strasser S. I. Berenguer M. (2017). Disease reversibility in patients with post-hepatitis c cirrhosis: Is the point of no return the same before and after liver transplantation? a review. Transplantation 101, 916–923. doi: 10.1097/TP.0000000000001633 28060241

[B30] Wang Q. Zhao H. Deng Y. Zheng H. Xiang H. Nan Y.. (2022). Validation of baveno VII criteria for recompensation in entecavir-treated patients with hepatitis b-related decompensated cirrhosis. J. Hepatol. 77, 1564–1572. doi: 10.1016/j.jhep.2022.07.037 36038017

[B31] Xu X. Wang H. Zhao W. Wang Y. Wang J. Qin B. (2021). Recompensation factors for patients with decompensated cirrhosis: a multicentre retrospective case-control study. BMJ Open 11, e043083. doi: 10.1136/bmjopen-2020-043083 PMC8230976 34162632

[B32] Zhao H. Wang Q. Luo C. Liu L. Xie W. (2020). Recompensation of decompensated hepatitis b cirrhosis: Current status and challenges. BioMed. Res. Int. 2020, 9609731. doi: 10.1155/2020/9609731 33029534 PMC7527887

