# Supplementary material for: Ascites re-compensation in HBV-related first decompensated cirrhosis after anti-viral therapy
Source: Front Cell Infect Microbiol. 2023 Jan 12;12:1053608. doi: 10.3389/fcimb.2022.1053608 (PMC9878306; doi:10.3389/fcimb.2022.1053608)
Supplement: Supplementary file 1 [file DataSheet_1.docx]

**
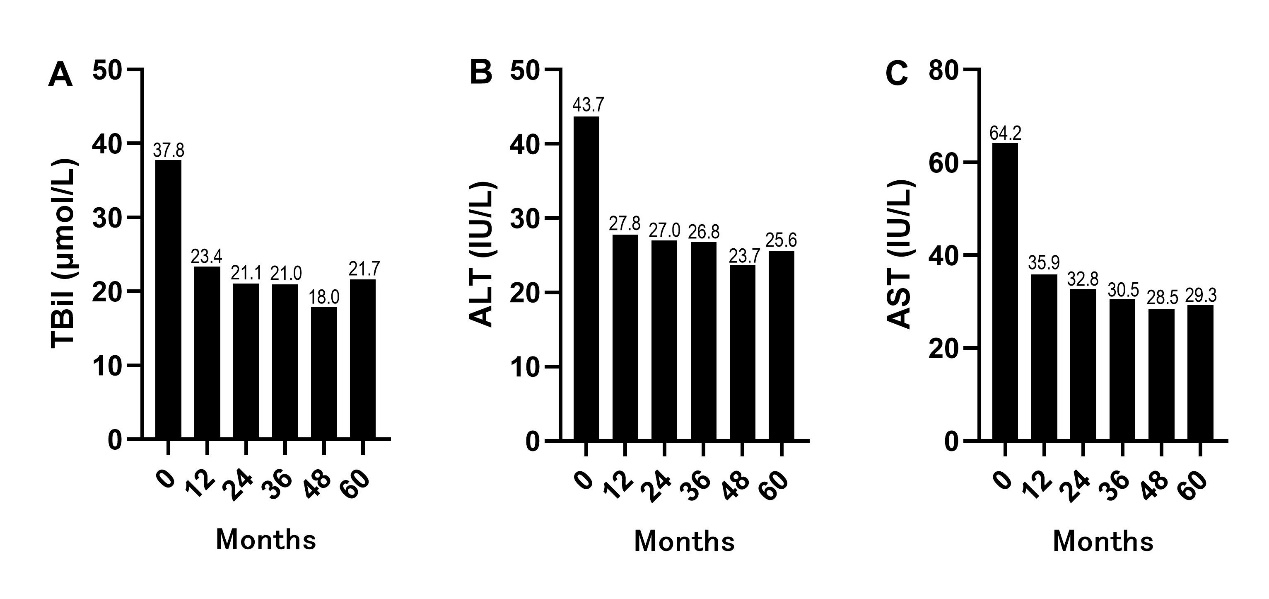
FIGURE S1 The dynamic change of TB, ALT and AST after antiviral treatment at baseline, 12, 24, 36, 48 and 60 months in patients with first decompensated HBV cirrhosis of ascites.** (A) Changes of TB in patients after NUC treatment at baseline, 12, 24, 36, 48 and 60 months; (B) Changes of ALT in patients after NUC treatment at baseline, 12, 24, 36, 48 and 60 months; (C) Changes of AST in patients after NUC treatment at baseline, 12, 24, 36, 48 and 60 months.


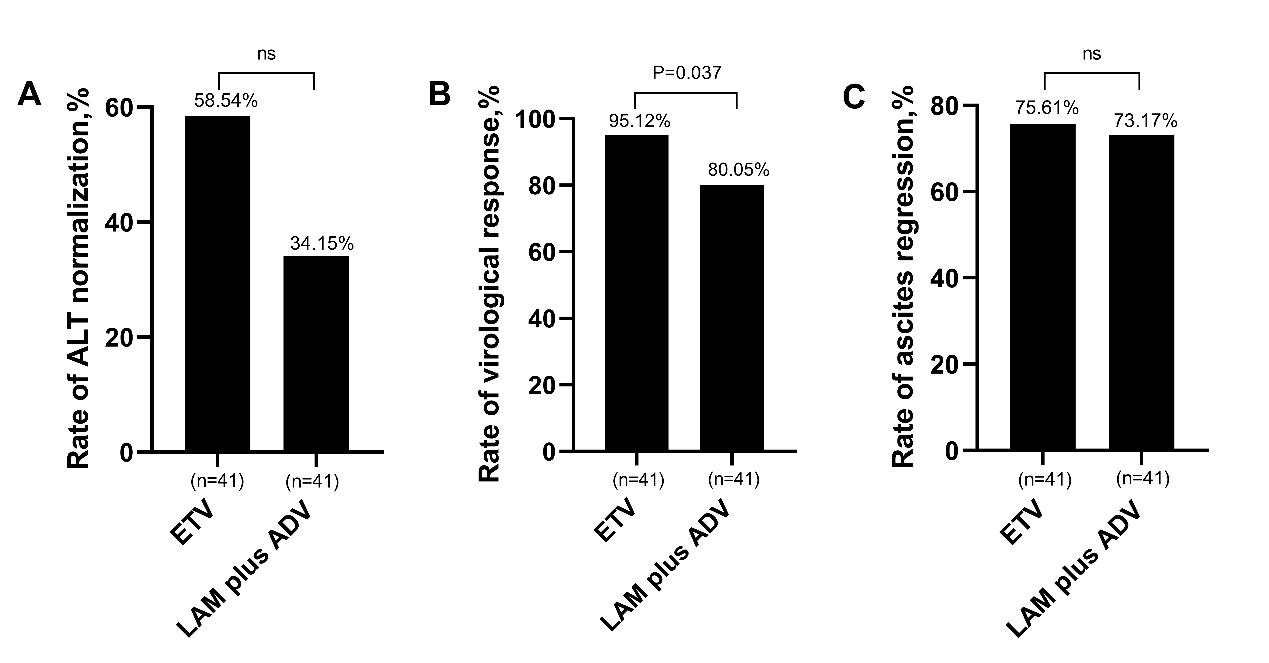


**FIGURE S2 Comparison of the virological response rate, biochemical response rate and ascites regression rat**e **between ETV monotherapy group and LAM and ADV combination therapy group.** (A) Comparison of the ALT normalization rate between ETV monotherapy group and LAM and ADV combination therapy group; (B) Comparison of the virological response rate between ETV monotherapy group and LAM and ADV combination therapy group; (C) Comparison of the ascites regression rate between ETV monotherapy group and LAM and ADV combination therapy group.

**Table S1 Virological response and biochemical response of different NUC at 12-month in the treatment of patients with hepatitis B decompensated cirrhosis**

|  | ETV  (n=152) | | LAM plus ADV  (n=41） | TDF  (n=1) | TAF  (n=1) | | LdT plus ADV  (n=1） |
| --- | --- | --- | --- | --- | --- | --- | --- |
| Patients of virological response, n(%) | 142  (93.42%) | 32(78.05%)* | | 1(100%) | 1(100%) | 1(100%) | |
| Patients of biochemical response, n(%) | 94  (61.84%) | 17(41.46%)* | | 1(100%) | 1(100%) | 0(0%) | |

Compared with ETV therapy, *P<0.05

Abbreviations: ETV, entecavir; LAM plus ADV, lamivudine plus adefovir dipivoxil; LdT plus ADV, tebivudine plus adefovir dipivoxil; TDF, tenofovir fumarate; TAF, tenofovir alafenamide fumarate.

**Table S2** **Baseline characteristics of the re-compensation of ascites and no re-compensation of ascites patients**

| Variables | re-compensation of ascites (=114) | | no re-compensation of ascites (n=82) | P value |
| --- | --- | --- | --- | --- |
| Age, y | | 50.1±10.5 | 54.5±9.5 | 0.829 |
| Male, n | | 79(69.3) | 58(70.7) | 0.394 |
| ascites | |  |  | 0.005 |
| grade 1 | | 98(86.0) | 57(69.5) |  |
| grade 2 and grade 3 | | 16(14.0) | 25(30.5) |  |
| ETV therapy, n | | 94(82.5) | 58(70.7) | 0.081 |
| TBIL, μmol/L | | 46.5(30.2-82.4) | 28.1(19.6-44.8) | 0.015 |
| ALT, IU/L | | 63.2(36.8-210.5) | 36.2(26.5-46.9) | 0.454 |
| AST, IU/L | | 88.8(50.2-210.7) | 53.3(36.8-70.8) | 0.349 |
| HBV DNA, log_10_ IU/mL | | 5.0(3.0-6.0) | 5.0(3.0-6.0) | 0.232 |
| Child-Pugh class | |  |  | 0.013 |
| A | | 3(2.6) | 1(1.2) |  |
| B | | 44(38.6) | 49(59.8) |  |
| C | | 67(58.8) | 32(39.0) |  |
| MELD score | | 12.0(9.0-17.0) | 9.0(7.0-13.0) | 0.022 |

Abbreviations: ETV, entecavir; TBIL, total bilirubin; ALT, alanine aminotransferase; AST, aspartate aminotransferase; MELD, model for end-stage liver disease.
